# Bioinformatic analysis of KIT juxtamembrane domain mutations in Syrian GIST patients: jigsaw puzzle completed

**DOI:** 10.1186/s43046-023-00185-0

**Published:** 2023-08-14

**Authors:** Nour Pharaon, Wafa Habbal, Fawza Monem

**Affiliations:** 1 Department of Biochemistry and Microbiology, Faculty of Pharmacy, Damascus University, Damascus, Syria; 2 Clinical Laboratories Department, Al-Assad Hospital, Damascus University, PO Box 10769, Damascus, Syria

**Keywords:** Bioinformatics, Exon 11, GIST, Juxtamembrane, *KIT*, Mutations, Syria

## Abstract

**Background:**

The huge number of detected somatic *KIT* mutations highlights the necessity of in silico analyses that are almost absent in the relevant medical literature. The aim of this study is to report the mutation spectrum analysis of exon 11 encoding the juxtamembrane (JM) domain of the *KIT* gene in a group of Syrian GIST patients.

**Methods:**

Forty-eight formalin-fixed paraffin-embedded GIST tissue samples, collected between 2006 and 2016, were retrieved from the pathological archives and analyzed for *KIT* exon 11 mutations by DNA sequencing. Structural/functional impact of detected variants was predicted using several bioinformatic tools.

**Results:**

Twenty-one different variants have been detected in intron 10, exon 11, and intron 11 of the *KIT* gene, eight of which were novel changes. Mutations in exon 11 of the *KIT* gene were detected in 28 of 48 (58.3%) GIST patients and predicted to be pathogenic and cancer promoting. Specifically, age above 60 was very significantly associated with the negative selection of deletion mutations (*p* = .007), a phenomenon that points to deletion severity.

**Conclusions:**

Six bioinformatic tools have proved efficient in predicting the impact of detected *KIT* variations in view of published structural, experimental, and clinical findings.

## Background

Gastrointestinal stromal tumor (GIST) is the most common sarcoma in the GI tract and is distinguished from other mesenchymal tumors by CD117 (KIT) [1, 2]. This differential diagnostic immunohistochemical marker belongs to class III receptor tyrosine kinases (RTKs) characterized by extracellular, transmembrane, juxtamembrane, and bilobed kinase domains. The extracellular portion comprises five immunoglobulin-like domains involved in ligand binding and receptor dimerization, whereas the intracellular portion encompasses a ~ 30 amino-acid long regulatory juxtamembrane region and a kinase domain interposed by a ~ 80 amino-acid long kinase insert sequence [3, 4]. Ligand binding leads to receptor dimerization and tyrosine autophosphorylation in the juxtamembrane, kinase insert, and C-terminal tail, hence initiating signal transduction cascades and inducing cell growth and proliferation [3, 5].

CD117 is encoded by the *KIT* gene mapped to the chromosome region 4q12 and composed of 21 exons [6]. In GIST cases, *KIT* mutations most commonly cluster in exon 11 encoding the regulatory juxtamembrane (JM) domain of CD117 [3]. In the absence of ligand, wild-type JM domain inhibits KIT receptor dimerization and suppresses its kinase activity [3, 4]. Upon ligand binding, however, JM domain phosphorylation disrupts such autoinhibition and allows receptor activation [4]. Alternatively, mutations in exon 11 release JM suppression, induce ligand-independent constitutive receptor activation, and trigger uncontrolled cell division and carcinogenesis [3, 7].

The underlying biological changes in the receptor structure and function due to the *KIT* mutations as well as their impact on prognosis and therapeutic response have been enormously of interest to experimental and clinical studies [1, 8, 9]. Yet, the huge number of somatic *KIT* mutations that exceeded 2500 variants detected in more than 6000 GIST unique samples [6] precluded their individual analysis and highlighted the necessity of in silico analyses that are almost absent in the relevant medical literature. In this premier study within the Greater Middle East, we report the mutation spectrum of *KIT* exon 11, encoding the JM domain, in a group of Syrian GIST patients. Several bioinformatic tools have then been utilized to assess mutations pathogenicity in view of the structural, experimental, and clinical knowledge established in previous publications.

## Methods

### Specimens

All formalin-fixed paraffin-embedded GIST treatment-naïve tissue samples, collected from stomach, small or large intestine, or omentum and mesentery between 2006 and 2016, were retrieved from the pathological archives at Al Assad Hospital, Damascus University. Histopathological features such as cell type, mitotic rate, tumor grade, and immunohistochemical markers (CD117, CD34) were re-evaluated by two accredited pathologists. Areas containing more than 80% tumor cells were marked according to the hematoxylin–eosin (HE) staining and dissected by disposable sterile scalpels. The study was approved by the Research Ethics Committee of Damascus University, and written informed consent had already been obtained upon admission from all enrolled patients.

### Direct sequencing

Genomic DNA was extracted using a Dual Genomic DNA Isolation Kit-Tissue (GeneDireX, Inc., Taiwan) according to the manufacturer’s instructions. Protocol modifications included using proteinase K (Thermo Fisher Scientific, USA) for tissue lysis and skipping the phase-separation step. DNA extracts quality was assessed using the NanoDrop ND-1000 spectrophotometer (NanoDrop Technologies, Inc., USA).

Exon 11 and ~ 60-bp flanking intronic regions of the *KIT* gene were amplified and sequenced using a forward primer 5′-CCA GAG TGC TCT AAT GAC TGA GA-3′ and a reverse primer 5′-AAA CAA AGG AAG CCA CTG GA-3′ (Eurofins Genomics, Germany) [10]. The final 50-μL PCRs included 0.2 μM of each primer, 25-μL HotStar PCR SuperMix (GeneDireX, Inc., Taiwan), and 5 μL of DNA extract. Thermal cycling was initiated at 94 °C for 2 min followed by 45 cycles of denaturation at 94 °C for 30 s, annealing at 54 °C for 30 s, and extension at 72 °C for 1 min; a final extension at 72 °C for 7 min was added. The 281-bp PCR amplicons were visualized on 2.5% agarose gel, purified using a High Pure PCR Product Purification Kit (Roche Diagnostics, Germany), and sequenced using a BigDye Terminator v3.1 Cycle Sequencing Kit on the ABI PRISM® 3100-*Avant*™ Genetic Analyzer (Applied Biosystems, USA).

### Bioinformatic analysis

Exon and intron variants were identified, checked in the COSMIC [6], Ensembl [11], dbSNP [12], SNPedia [13], and LOVD [14] databases, and described according to the Human Genome Variation Society (HGVS) recommendations for Sequence Variant Nomenclature at the DNA and protein levels in relation to the NCBI Reference Sequence NG_007456.1 [15]. Structural/functional impact of all coding and noncoding variants were predicted using SIFT [16], PolyPhen-2 [17], fathmm [18], fathmm-MKL [19], AASsites [20], and MutationTaster [21] tools.

### Statistical analysis

Statistical analysis was performed using web-based GraphPad QuickCalcs (https://www.graphpad.com/quickcalcs/; accessed Oct 2022). Associations between demographic, histopathological, and molecular variables were analyzed using Fisher’s exact test. *P*-value < 0.05 was considered statistically significant.

## Results

### Demographic and histopathological characteristics

Among 48 GIST patients included in our study, 29 (60.4%) patients were male, and 19 (39.6%) were female. The mean age at diagnosis was 52.6 years, ranging from 17 to 81 years. Most tumor cases originated from the stomach (27, 56.3%) followed by small intestine (15, 31.3%), omentum and mesentery (4, 8.3%), and large intestine (2, 4.2%). Microscopic evaluation revealed that most tumors had spindle cell type (27, 56.3%) followed by epithelioid type (12, 25%) and mixed spindle and epithelioid type (9, 18.8%). Mitotic rate was low (≤ 5/50 HPFs) in 8 (16.7%) cases, moderate (6–10/50 HPFs) in 25 (52.1%) cases, and high (> 10/50 HPFs) in 15 (31.3%) cases. Tumor grade was high in 40 (83.3%) cases and low in 8 (16.7%) cases. Among 48 CD117-positive tumors, 5 (10.14%) were focal, and 43 (89.6%) were diffuse, while CD34 was positive in 30 (62.5%) cases (Table 1).

**Table 1:** Demographics and histopathological characteristics of Syrian GIST patients with mutations in exon 11 of the *KIT* gene (*n* = 28)

Case no	Histopathological characteristics			Molecular variations		Demographics	
	**Tumor grade**	**Mitotic rate**	**Cell morphology**	**Exon 11**	**Introns 10/11**	**Age (year)**	**Gender**
***Anatomic site: stomach***							
1	High	High	Epithelioid	p.(Trp557_Lys558del)		59	M
2	High	High	Mixed	p.(Trp557_Val559delinsPhe)		48	M
3	High	High	Mixed	p.(Tyr553_Val559del)		55	M
4	High	High	Mixed	p.(Lys550Arg)		71	M
5	High	High	Spindle	p.(Trp557_Lys558del)	**c.1774 + 5 G > A**^**a**^	55	M
6	High	High	Spindle	p.(Trp557_Val559delinsPhe)		61	M
7	High	Moderate	Epithelioid	**p.(Tyr553His)**^**a**^ **p.(Lys581Glu)**^**a**^		81	F
8	High	Moderate	Mixed	p.(Pro551_Val569delinsLeu)^b^		67	M
9	High	Moderate	Spindle	p.(Trp557_Lys558del)^b^ p.(Glu561Lys)^b^	**c.1648–29 G > A**^**ab**^ **c.1648–27 G > A**^**ab**^ **c.1774 + 9 G > A**^**ab**^	57	M
10	High	Moderate	Spindle	p.(Trp557_Lys558del) p.(Lys550Arg)		52	F
11	High	Moderate	Spindle	p.(Trp557_Lys558del)		63	M
12	Low	Low	Epithelioid	p.(Trp557_Lys558del) p.(Lys550Arg)		32	F
13	Low	Low	Epithelioid	p.(Trp557_Lys558del) p.(Lys550Arg)		59	M
14	Low	Low	Spindle	p.(Pro551_Glu554del) p.(Lys550Arg)		53	M
***Anatomic site: small intestine***							
15	High	High	Epithelioid	p.(Trp557_Lys558del) p.(Lys550Arg)		73	F
16	High	High	Epithelioid	p.(Lys550Arg)^b^		66	F
17	High	High	Spindle	p.(Trp557_Lys558del) p.(Lys550Arg)		50	F
18	High	High	Spindle	p.(Trp557_Lys558del) p.(Lys550Arg)		37	F
19	High	Moderate	Mixed	p.(Gly565Glu) p.(Arg586Lys)	**c.1774 + 37del**^**a**^	66	M
20	High	Moderate	Mixed	p.(Trp557_Lys558del)^b^ p.(Val555Ile)	**c.1774 + 5 G > A**^**a**^	54	F
21	High	Moderate	Mixed	p.(Lys550Arg)		44	F
22	High	Moderate	Spindle	p.(Trp557_Lys558del) p.(Lys550Arg)		49	F
23	High	Moderate	Spindle	p.(Trp557_Lys558del) p.(Lys550Arg)		76	M
24	High	Moderate	Spindle	p.(Trp557Gly)		66	F
25	Low	Low	Spindle	p.(Lys558_Val559del)		30	M
26	Low	Low	Spindle	**p.(Tyr570ThrfsTer2)**^**a**^		28	F
***Anatomic site: large intestine***							
27	High	High	Spindle	p.(Leu576Pro)		71	F
***Anatomic site: omentum and mesentery***							
28	Low	Low	Spindle	p.(Trp557_Lys558del)		48	M

^a^Variation is novel. ^b^Wild type not detected

### Molecular variations

Mutations in exon 11 of the *KIT* gene were detected in 28 of 48 (58.3%) GIST patients, four of whom (14.3%) had additional variations in intron 11 and/or intron 10 (Table 1). Exon 11 mutations included one deletion in 10 (35.7%) patients, one deletion and one substitution in 11 (39.3%) patients, and one or two substitutions in 7 (25%) patients. No duplications or insertions were encountered. Introns 10 and 11 variations included one deletion in 1 of 4 patients and one or three substitution(s) in 3 of 4 patients (Table 1).

Overall, twenty-one different variants have been detected in intron 10, exon 11, and intron 11 of the *KIT* gene, eight of which were novel changes. Exon variations, mostly affecting codons 553–559, included nine single-nucleotide/amino acid substitutions (Table 2) as well as seven 1-bp, 6-bp, 12-bp, 21-bp, or 57-bp deletions leading to amino acid deletions, indels, or frameshift changes (Table 3). Intron single-nucleotide variations included four substitutions and one deletion (Table 4).

**Table 2:** Substitutions detected in exon 11 of the *KIT* gene in Syrian GIST patients and in silico prediction of their effect on protein structure and function

Genomic change	CDS change	Protein change	Frequency	SIFT^b^	PolyPhen-2^c^	fathmm^d^	MutationTaster
g.74423A > G g.74424A > G	c.1649A > G c.1650A > G	p.(Lys550Arg)	12	Deleterious (0)	Probably damaging (0.996)	Cancer promoting (− 6.07)	Disease causing (0.999)^f^
**g.74431T > C**^**a**^	**c.1657 T > C**	**p.(Tyr553His)**	1	Deleterious (0)	Probably damaging (1)	Cancer promoting (− 6.63)	Disease causing (0.999)
g.74437G > A	c.1663G > A	p.(Val555Ile)	1	Tolerated (0.67)	Benign (0.039)	Cancer promoting (− 5.68)	Disease causing (0.999)
g.74443T > G	c.1669T > G	p.(Trp557Gly)	1	Deleterious (0)	Probably damaging (1)	Cancer promoting (− 6.26)	Disease causing (0.999)
g.74455G > A	c.1681G > A	p.(Glu561Lys)	1	Deleterious (0)	Possibly damaging (0.875)	Cancer promoting (− 6.39)	Disease causing (0.999)
g.74468G > A	c.1694G > A	p.(Gly565Glu)	1	Deleterious (0)	Probably damaging (1)	Cancer promoting (− 7.20)	Disease causing (0.999)
g.74501T > C	c.1727T > C	p.(Leu576Pro)	1	Deleterious (0)	Possibly damaging (0.627)	Cancer promoting (− 6.92)	Disease causing (0.999)
**g.74515A > G**^**a**^	**c.1741 A > G**	**p.(Lys581Glu)**	1	Deleterious (0.01)	Possibly damaging (0.811)	Cancer promoting (− 6.29)	Disease causing (0.999)
g.74531G > A	c.1757G > A	p.(Arg586Lys)	1	Deleterious (0)	Possibly damaging (0.88)	Passenger^e^ (− 1.74)	Disease causing (0.999)

^a^Variation is novel. ^b^SIFT: 0 ≤ score < 0.05, deleterious; 0.05 < score ≤ 1, tolerated. ^c^PolyPhen-2: 0 ≤ score ≤ 0.14, benign; 0.15 ≤ score ≤ 0.84, possibly damaging; 0.85 ≤ score ≤ 1, probably damaging. ^d^fathmm: score <  − 3, cancer promoting; score >  − 3, passenger. ^e^ “Cancer promoting” when the less stringent default prediction threshold (− 0.75) was used. ^f^Prediction probability, a value close to 1 indicates a high “security” of the prediction

**Table 3:** Deletions detected in exon 11 of the *KIT* gene in Syrian GIST patients and in silico prediction of their effect on protein structure and function

Genomic change	CDS change	Protein change	Frequency	MutationTaster
g.74425_74436del12	c.1651_1662del12	p.(Pro551_Glu554del)	1	Disease causing (0.947)^b^
g.74426_74479del54	c.1652_1705del54	p.(Pro551_Val569delinsLeu)	1	Disease causing (0.999)
g.74431_74451del21	c.1657_1677del21	p.(Tyr553_Val559del)	1	Disease causing (0.999)
g.74443_74448del6	c.1669_1674del6	p.(Trp557_Lys558del)	14	Disease causing (0.999)
g.74444_74449del6	c.1670_1675del6	p.(Trp557_Val559delinsPhe)	2	Disease causing (0.986)
g.74446_74451del6	c.1672_1677del6	p.(Lys558_Val559del)	1	Disease causing (0.999)
**g.74480delT**^**a**^	**c.1706delT**	**p.(Tyr570ThrfsTer2)**	1	Disease causing (1)

^a^Variation is novel. ^b^Prediction probability, a value close to 1 indicates a high “security” of the prediction

**Table 4:** Novel variations detected in introns 10 and 11 of the *KIT* gene in Syrian GIST patients and in silico prediction of their effect on protein structure and function

Genomic change	CDS change	Frequency	fathmm-MKL^a^	MutationTaster	AASsites^c^
g.74553G > A	c.1774 + 5G > A	2	Pathogenic (0.98)	Disease causing (1)^**b**^	Likely change
g.74557G > A	c.1774 + 9G > A	1	Neutral (0.42)	Polymorphism (0.999)	No change
g.74393G > A	c.1648-29G > A	1	Neutral (0.3)	Polymorphism (0.999)	No change
g.74395G > A	c.1648-27G > A	1	Neutral (0.29)	Polymorphism (0.999)	No change
g.74585delT	c.1774 + 37del	1	NA	Polymorphism (0.999)	NA

^a^fathmm-MKL: score < 0.5, neutral; score > 0.5, pathogenic; NA, non-applicable. ^b^prediction probability, a value close to 1 indicates a high 'security' of the prediction. ^c^Likely change, variation changes splicing pattern; no change, variation has no effect on splicing pattern; NA, non-available, test not performed as the tool was not accessible anymore

### Bioinformatic analysis

Most substitutions detected in the JM domain of the *KIT* gene in our GIST patients were predicted to have deleterious (SIFT; 0) and probably/possibly damaging (PolyPhen-2; 0.627–1) effect on protein structure and function and to be cancer promoting (fathmm; <  − 6.00) and disease causing (MutationTaster; probability 0.999; Table 2). However, in silico predictions obtained for p.(Val555Ile) and p.(Arg586Lys) substitutions were discordant. In addition, all deletions detected in the JM domain of the *KIT* gene in our GIST patients were predicted to be disease causing (MutationTaster; probability ≥ 0.9; Table 3). Among intronic variations, only c.1774 + 5G > A was shown to be pathogenic (fathmm-MKL; 0.98), disease causing (MutationTaster; probability 1), and likely changing the splicing pattern (AASsites; Table 4).

### Statistical analysis

High tumor grade was significantly associated with age above 60 (*p* = 0.04) but independent of gender (*p* = 1.0), number of mutations (*p* = 1.0), and occurrence of deletions (*p* = 0.29) or any mutation (*p* = 0.44) in the JM domain of the *KIT* gene. Albeit not associated with gender (*p* = 0.54), the mutant status of the JM domain (*p* = 0.36), or number of JM domain mutations (*p* = 0.46), age above 60 was significantly associated with the partial absence of deletion mutations in favor of substitution mutations (*p* = 0.007). Contrariwise, gender, anatomic site, mitotic rate, and cell morphology were independent (*p* > 0.05) of the number of mutations and the occurrence of deletions or any mutation in the JM domain of the *KIT* gene.

## Discussion

Due to its essential role of suppressing KIT proto-oncoprotein, the juxtamembrane (JM) domain releases its brake, when mutated, leading to proliferation promotion and apoptosis inhibition [3, 7]. This involvement of the JM domain-encoding exon 11 in GIST development is highly revealed by an average 86% mutation rate among *KIT*-mutated GIST patients [22–25]. In this study, exon 11 was mutated in about 58% of GIST patients approximating the average worldwide rate (60%) that spreads over the range 22–90% in more than a hundred studies. In discord with inherited variations, these reported frequencies of exon 11 somatic mutations among GIST patients were not correlated with geographic region according to our statistical estimation (data not shown). Nucleotide deletions and substitutions were the only mutation types detected in our GIST patients (Table 1); this accords with the majority of publications where deletions and substitutions are considerably reported contrary to insertions and duplications [26, 27]. In discord with numerous studies [2, 28], however, the combination of deletion-substitution mutation type was highly detected in our study group (Table 1).

Our bioinformatic analysis by SIFT, PolyPhen-2, fathmm, and MutationTaster tools revealed that all seven deletions and nine amino acid substitutions encountered in the JM domain were pathogenic (Tables 2 and 3). Despite their sole discordant predictions between the four tools, we suppose that Val555Ile and Arg586Lys mutations are more likely pathogenic according to the pathogenicity weight, conservation index, and large diverse databases adopted by relevant tools. Hence, the occurrence of any of these sixteen mutations in the *KIT* proto-oncogene might promote tumorigenesis. Their pathogenicity is further emphasized as high tumor grade seemed independent of type and number of mutations encountered in the JM domain of the *KIT* gene in our GIST patients (*p* > 0.05).

This virtual outcome is underlined by X-ray crystallographic reports that demonstrated the key role of the JM domain in general and its critical codons Tyr^553^, Trp^557^, Val^559^, Val^560^, and from Tyr^547^ to Gly^565^ in particular [29]. Notably, these aforementioned codons were affected by most presumably pathogenic mutations detected in our GIST patients, possibly disrupting JM function in stabilizing autoinhibited KIT and leading to cancer development [30, 31]. Moreover, the severity of these gain-of-function mutations in the JM domain, whether being substitutions or deletions, has already been manifested by in vitro and in vivo experimental evidence [30, 31]. Besides folding autonomously, the JM domain has been found to be highly conserved in class III receptor tyrosine kinases (RTKs) family. In fact, mutant JM domain demonstrated disordered secondary structure, reduced binding affinity to the kinase domain, less effective inhibition of receptor catalytic activity, and extremely rapid kinetics of ligand-independent receptor activation. Ultimately, the existence of substitution or deletion mutations in the JM domain of the KIT protein seemed sufficient for cell transformation [31].

Obviously, deletion mutations were specifically severe and seem to lower survival rate; this is supported by the following:

- (i) Their high frequency in our cases with mutant JM domain (21 of 28; 75%).
- (ii) Their apparent negative selection in favor of substitution mutations in our elderly GIST patients.
- (iii) In addition to clinical evidence inferred by more than a dozen studies [8, 32, 33]

Interestingly, Trp557_Lys558del mutation was the most frequently encountered deletion (14 of 21; 67%). Furthermore, deletion mutations affected both codons 557 and 558 in 90% (19 of 21) of our cases. These observations point to the phenomenal severity of deletions in these amino acid positions.

GIST patients with mutated JM domain have significantly higher response rates to imatinib therapy [1, 9, 34, 35]; this was evident in seven of our patients including four responders with exon 11 mutations and three nonresponders with wild-type exon 11. Nevertheless, therapy information for the remaining GIST patients was not retrievable; hence, the association between imatinib response and the JM domain mutations could not be analyzed.

Our data indicated an association between high tumor grade and age above 60 (*p* < 0.05), an observation likely attributed to the accumulation of additional mutations over a longer lifespan in other loci of the genome. This explains the absence of association between tumor grade and the occurrence of JM domain mutations in our GIST patients (*p* > 0.05). Obviously, gender might not contribute to the tumor pathogenicity as it appeared independent of the occurrence, type, and number of JM domain mutations as well as tumor grade and age of onset. Nevertheless, it has been previously observed that men are slightly more likely to develop the disease and to have a worse prognosis than women [36, 37].

Extending the catalogue of *KIT* mutations in GIST, eight novel additional variations have been detected in our patients including two in intron 10, three in exon 11, and three in intron 11. Intriguingly, the two novel substitutions in Tyr^553^ and Lys^581^ occurred in one case and were deemed pathogenic by all utilized bioinformatic tools. This consists with the significance of Tyr^553^ amino acid in inhibiting KIT by interacting with Asp^810^ and Glu^640^ of kinase domain [29]. The third novel virtually pathogenic single-nucleotide deletion in exon 11 shifted the reading frame and incorporated a premature stop codon perhaps leading to the production of a truncated protein. The novel intron variations were supposedly neutral via in silico analysis except the one encountered twice and located within a splicing motif. These findings might not be conclusive as enrolling healthy individuals was beyond the scope of our study. Further biological and clinical studies might reveal the impact of these novel variations. Otherwise, genome-wide association studies in the future might classify such presumably neutral variants as polymorphisms.

## Conclusions

In this study, we reported the mutation spectrum analysis of *KIT* exon 11, encoding the JM domain, in a group of Syrian GIST patients, where three supposedly pathogenic novel mutations were uncovered. Six bioinformatic tools have proved efficient in predicting the impact of detected *KIT* variations in view of published structural, experimental, and clinical findings. Utilizing computational analysis as a start point to guide research studies seems helpful to save human, financial, and material resources.

## Data Availability

The datasets used and/or analyzed during the current study are available from the corresponding author on reasonable request.

## Abbreviations

GIST: Gastrointestinal stromal tumor
HE: Hematoxylin-eosin
HGVS: Human Genome Variation Society
HPF: High-power fields
JM: Juxtamembrane
RTK: Receptor tyrosine kinase
